# Regulation of Epithelial Cell Morphology and Functions Approaching To More In Vivo-Like by Modifying Polyethylene Glycol on Polysulfone Membranes

**DOI:** 10.1371/journal.pone.0036110

**Published:** 2012-04-27

**Authors:** Chong Shen, Guoliang Zhang, Qin Meng

**Affiliations:** 1 Department of Chemical and Biological Engineering, Zhejiang University, Hangzhou, China; 2 College of Biological and Environmental Engineering, Zhejiang University of Technology, Hangzhou, China; National Cancer Institute, United States of America

## Abstract

Cytocompatibility is critically important in design of biomaterials for application in tissue engineering. However, the currently well-accepted “cytocompatible" biomaterials are those which promote cells to sustain good attachment/spreading. The cells on such materials usually lack the self-assembled cell morphology and high cell functions as in vivo. In our view, biomaterials that can promote the ability of cells to self-assemble and demonstrate cell-specific functions would be cytocompatible. This paper examined the interaction of polyethylene glycol (PEG) modified polysulfone (PSf) membranes with four epithelial cell types (primary liver cells, a liver tumor cell line, and two renal tubular cell lines). Our results show that PSf membranes modified with proper PEG promoted the aggregation of both liver and renal cells, but the liver cells more easily formed aggregates than the renal tubular cells. The culture on PEG-modified PSf membranes also enhanced cell-specific functions. In particular, the cells cultured on F127 membranes with the proper PEG content mimicked the in vivo ultrastructure of liver cells or renal tubules cells and displayed the highest cell functions. Gene expression data for adhesion proteins suggest that the PEG modification impaired cell-membrane interactions and increased cell-cell interactions, thus facilitating cell self-assembly. In conclusion, PEG-modified membrane could be a cytocompatible material which regulates the morphology and functions of epithelial cells in mimicking cell performance in vivo.

## Introduction

Human tissues and organs are organized by the interactions of individual cells with each other and with extracellular matrix (ECM) [1]. In this regard, the ECM has been the model for developing synthetic biomaterials for tissue engineering, drug delivery, medicine, and biotechnology [2], [3]. As such biomaterials generally need to contact cells or tissues in applications, it is extremely important that they are cytocompatible, i.e., that they generate “the most beneficial cellular response" [2], [3].

To achieve cytocompatible synthetic biomaterials, the regulatory characteristics of tissue and organ ECM have been mimicked by introducing defined molecular-recognition elements [4], [5]. Among these elements, the most frequently reported include grafting the integrin-binding arginine-glycine-aspartic acid (RGD) sequence [5], which is abundant in many ECM proteins, growth factors (e.g., hepatocyte growth factor and fibroblast growth factor-2) [6], and receptor-binding molecules (e.g., galactose for hepatocytes [7]). Nevertheless, these recognition molecules are structurally sophisticated and chemically unstable, so that using such elements to modify the surface of biomaterials normally increases their complexity [5]. Hence, an alternative proposal to improve the cytocompatibility of surfaces has been to fabricate biomaterials with simpler structures, either by modifying their surface topography or hydrophilicity [5], [8]. The topography of biomaterials was modified by a micropatterned array [9] or surface-roughness control [10], while their hydrophilicity was improved by grafting hydrophilic molecules such as acrylic acid [11] and 2-hydroxyethyl methacrylate [12].

Surface modification of biomaterials by either recognition elements or surface topography/hydrophilicity generally leads to a high rate of cell adhesion/spreading/proliferation, which has been well accepted as an index of cytocompatibility [13], [14], [15]. Hence, the cytocompatibility is currently assayed by the viability of attached/proliferating cells [13], [14], [15], which more likely reflects the non-cytotoxicity of biomaterials. In fact, well-attached/spreading cells on biomaterials usually proliferate at a high rate, but their functions are not well differentiated [1]. In contrast, anchor-dependent cells in vivo, which are supported by the endogenous ECM network, commonly show a low proliferation rate and high degree of differentiation [16]. For example, in either healthy liver tissue or liver tumors, highly organized cells (hepatocytes or liver tumor cells) are non- or low-proliferating [17] and loosely surrounded by the ECM, including collagen and fibronectin [18]. These functional cells in vivo, lacking a strong interaction with the ECM, organize into three-dimensional multicellular structures in tissues and organs, deviating from the high spreading/proliferation state found in vitro [16]. However, this in vivo aspect of cytocompatibility has rarely been the focus in designing synthetic biomaterials.

An important biomaterial used extensively in bioartificial organs, despite its poor cytocompatibility, is the polymeric membrane [19], [20], [21]. Such a membrane, polysulfone (PSf) membranes grafted with small polyethylene glycol (PEG, MW 350), was occasionally found to support the self-assembly of primary hepatocytes into spheroids and to promote the expression of higher liver-specific functions than the attached hepatocytes on unmodified membranes [22]. To systematically investigate this phenomenon, we prepared a series of flat ultrafiltration membranes by blending PSf membranes with Pluronics of varying PEG content and studied the effect of PEG content on cellular morphology and functions. Pluronics are PEG-polypropylene oxide (PPO)–PEG triblock copolymers that anchor firmly in the polymer matrix via hydrophobic PPO segments, thus modifying the membrane surface via free hydrophilic PEG segments [23]. The cytocompatibility of each membrane was evaluated by emphasizing both the self-assembly and function of epithelial cells represented by four cell types: primary rat hepatocyte, human hepatocellular carcinoma (HepG2), Madin-Darby canine kidney (MDCK) and human kidney-2 (HK-2) renal tubular cell lines.

## Results

### Increasing PEG content of membrane surface improves hydrophilicity

PEG-modified PSf membranes were first prepared by blending PSf with 20% (wt/wt) L121/P123/F127 (Table 1) to obtain a typical ultrafiltration structure with a dense skin layer and a finger-like macroporous section. PSf membranes with a higher Pluronic content (over 20%) had an overly porous structure and were excluded in the following studies.

**Table 1:** Formation of casting solutions of membranes.

*Membranes*	*Composition of casting solution*			*PEG content in membrane(wt%)*	*Contact angle* (°)
	*PSf (mg)*	*Pluronics(mg)*	*DMAc (ml)*		
PSf	180.0	0.0	1	0	87.1±2.5
L121	144.0	36.0		2	77.5±1.8
P123	144.0	36.0		6	76.0±3.5
F127	144.0	36.0		14	68.2±2.1

**Figure 1:**
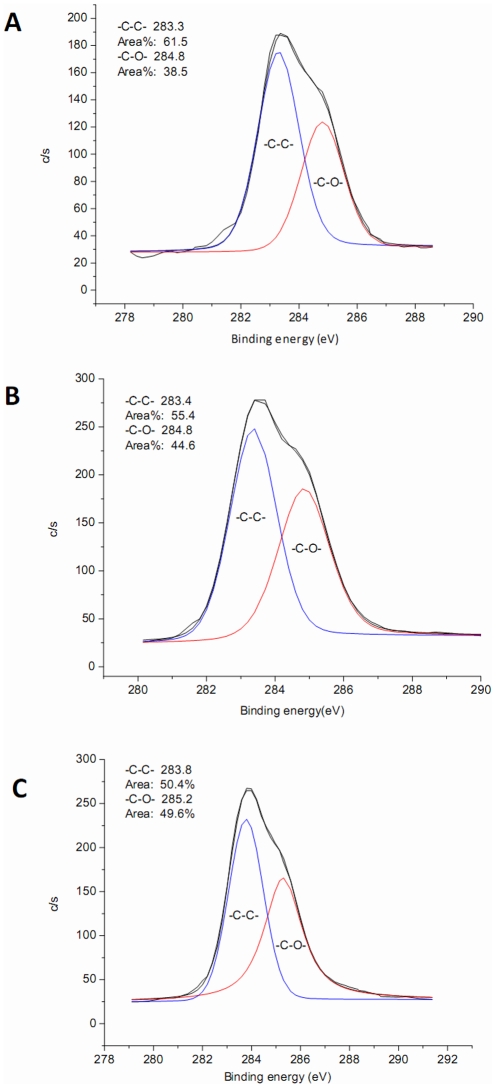
High-resolution X-ray photoelectron spectroscopy spectra of C 1 s for membranes. A: L121 membrane, B: P123 membrane, C: F127 membrane.

The PEG content of membrane surfaces was qualitatively assessed by XPS. The percent area of –C-O- bond on membranes was ranked as F127>P123>L121, indicating increasing PEG content on these membrane surfaces (Fig. 1). In addition, the sulfur signal was very weak on the three membrane surfaces (data not shown), indicating that the PSf membrane (containing sulfur) was almost completely covered by Pluronics (containing no sulfur). Correspondingly, the PEG modification significantly decreased the static water contact angle of membranes in this order F127<P123<L121<PSf (Table 1), indicating that Pluronic blending improved surface hydrophilicity.

It should be mentioned that the surface –C-O-/-C-C- ratio was not significantly altered by 1-month preservation in deionized water before being autoclaved at 121°C for 30 min, indicating negligible PEG flushing from membranes.

### Increasing PEG content of membrane surface decreases cell adhesion and proliferation

**Figure 2:**
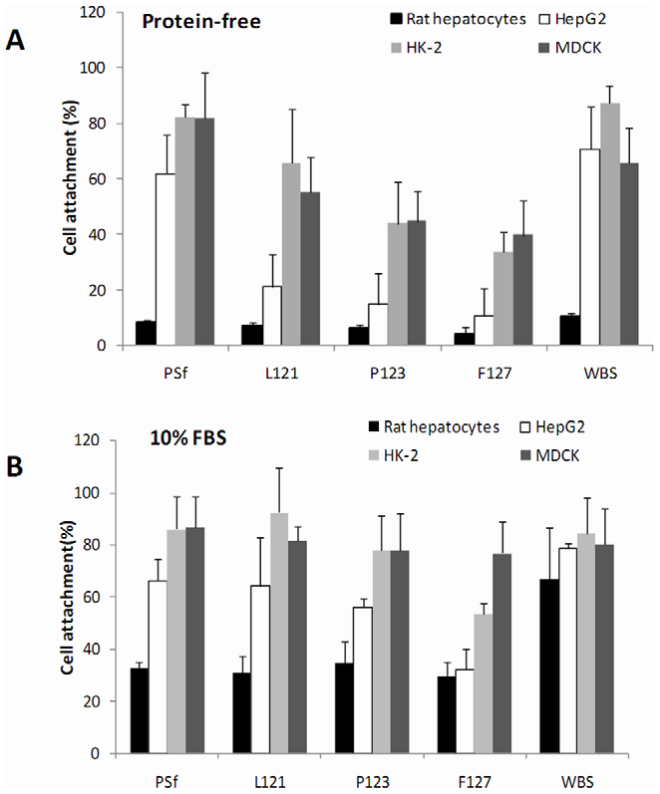
Cell attachment 4 h after seeding. A: in 10% FBS medium; B: in protein-free medium. Well bottom surfaces (WBS) were used as controls.

To demonstrate the role of serum proteins in cell adhesion, the four membranes were assessed for cell attachment at 4 h after seeding with primary cultured hepatocytes, HepG2, MDCK, and HK-2 cells in both FBS medium and protein-free medium (Fig. 2). Regardless of the membrane, primary cultures of rat hepatocytes displayed much lower adhesion in protein-free medium (Fig. 2A) than in FBS medium (Fig. 2B). The three cell lines showed similar adhesion in the two mediums on PSf membrane/WBS, but if cultured on PEG-modified membranes, had significantly lower adhesion in protein-free medium (Fig. 2A) than in FBS medium (Fig. 2B). All material surfaces generally showed acceptable cell adhesion in FBS medium, which is commonly used in cell culture, although cells on the F127 membrane had the lowest adhesion (Fig. 2B). We also noticed that cells were more weakly attached to PEG-modified membranes as they could be removed from these membranes by flushing with PBS, but not from the unmodified PSf membrane/WBS.

**Figure 3:**
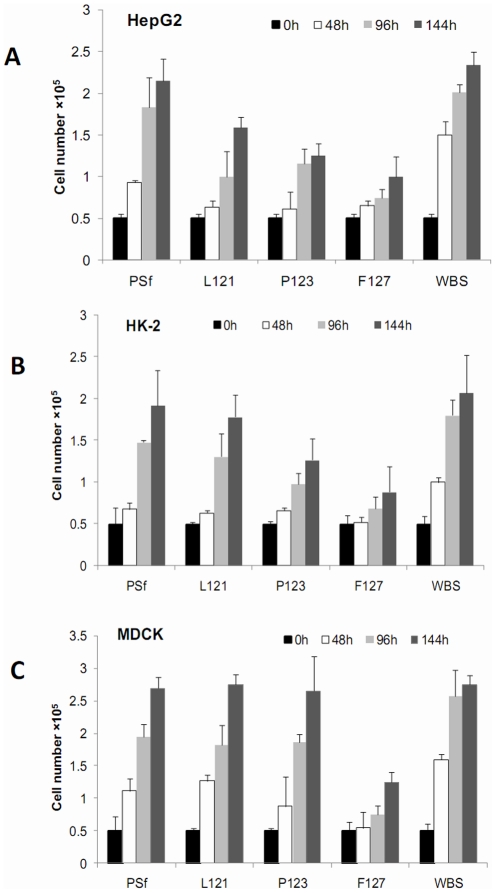
Cell proliferation on various membranes. Well bottom surfaces (WBS) were used as controls.

As expected, rat hepatocyte primary cultures did not proliferate in vitro (data not shown). HepG2 and HK-2 cells proliferated on membranes in the following descending order: WBS>PSf>L121>P123>F127 (Fig. 3A–B), while MDCK cells had a similar proliferation rate on all membranes except for the F127 membrane (Fig. 3C).

### Liver and renal tubular cells aggregate on membranes with high PEG content

**Figure 4:**
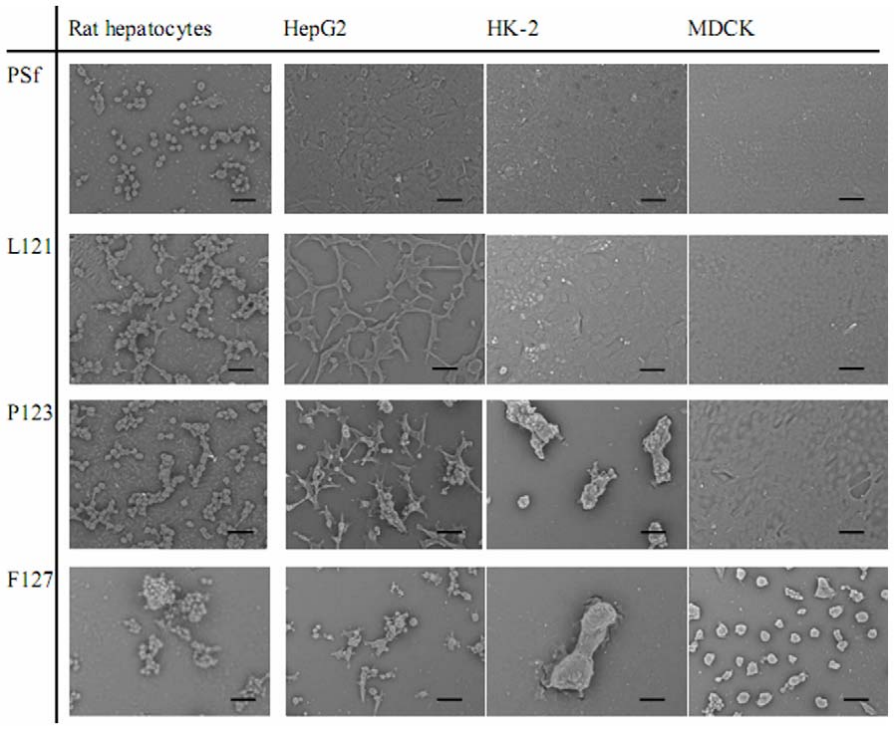
Morphology of HK-2, MDCK, and HepG2 cell lines and rat hepatocytes cultured for 144 h on various membranes. Cell images were detected by scanning electron microscope. Scale bar represents 50 µm.

Cell morphology after 144 h of culture on various membranes was assessed by SEM (Fig. 4). All four cell types clearly formed confluent monolayers on WBS (data not shown). Rat hepatocytes largely attached to PSf membranes, but aggregated on the remaining three membranes. Similarly, HepG2 liver cells displayed a flat and confluent morphology on PSf membranes, but showed elongated/flat aggregates on L121 membranes and round spheroids on P123/F127 membranes. HK-2 renal cells spread well to confluent layers on PSf and L121 membranes, but completely aggregated on P123 and F127 membranes. MDCK renal cells only formed aggregates on F127 membranes, and formed confluent layers on the other three membranes.

### Cell-specific functions of liver and renal tubular cells are higher when grown on PEG-modified membranes

**Figure 5:**
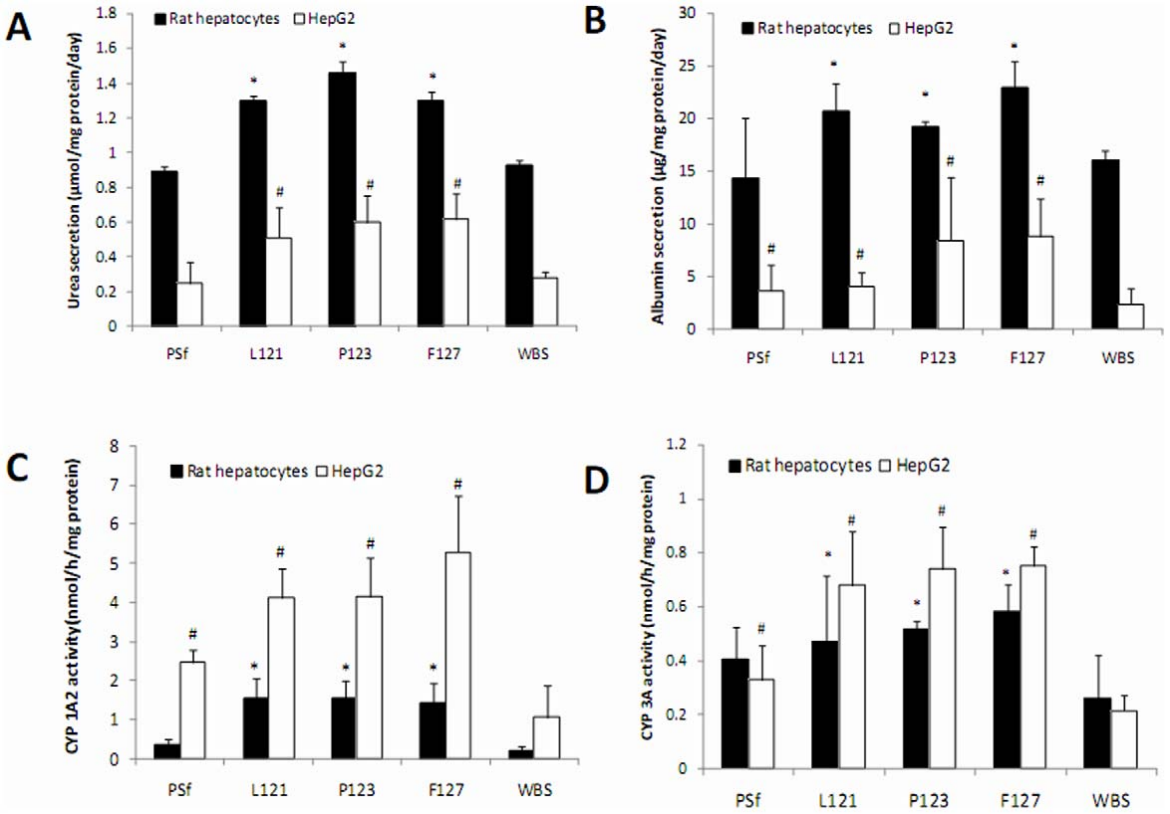
Liver-specific functions of rat hepatocytes and HepG2 cultured on various membranes. Urea production (A), albumin secretion (B), cytochrome P450 (CYP) 1A2 activity (C), and CYP 3A activity (D) are presented as means ± SD of three measurements. # and * represent significant differences (p<0.05) compared to the respective control on well bottom surfaces (WBS).

**Figure 6:**
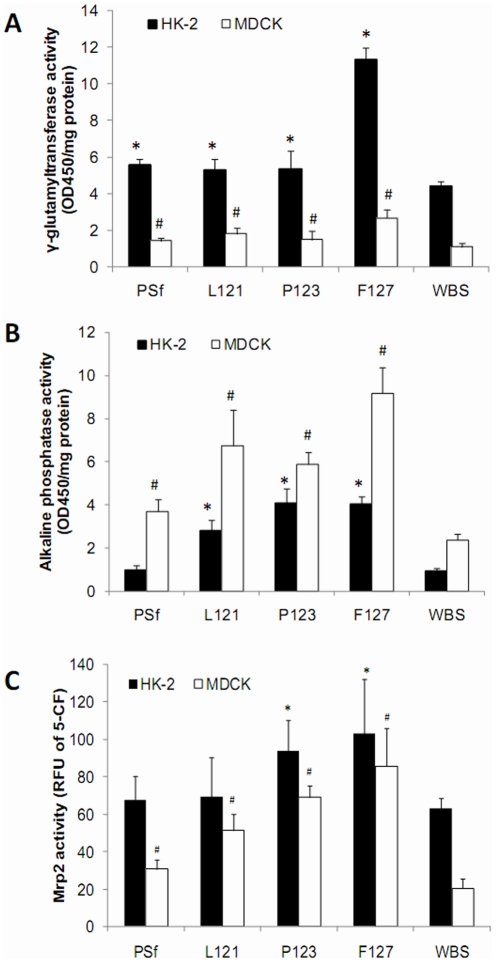
Renal tubular-cell specific functions of HK-2 and MDCK renal tubular cells cultured on various membranes. γ-glutamyltransferase activity (A), alkaline phosphatase activity (B), and Mrp2 activity (C) are presented as means ± SD of three measurements. # and * represent significant differences (p<0.05) compared to the respective control on well bottom surfaces (WBS).

To determine the effects of PEG modification on liver and renal tubular cells grown on membranes, liver and renal cell-specific functions were analyzed. Rat hepatocyte primary cell cultures and HepG2 cells grown on PEG-modified membranes had significantly higher urea/albumin secretion and cytochrome P 1A2/3A activity than those on PSf membrane and WBS (Fig. 5). HK-2 and MDCK cells exhibited the highest renal functions (γ-glutamyltransferase, alkaline phosphatase, and MRP2 activities) when grown on F127 membranes, followed by growth on P123 and L121 membranes (Fig. 6). In contrast, these cells had much lower renal functions on PSf membrane and WBS (Fig. 6).

### The cell-adhesion proteins and ultrastructure are influenced by culture on PEG-modified membranes

**Figure 7:**
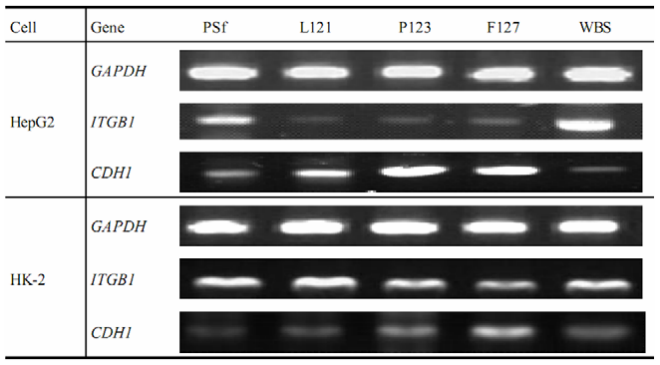
RT-PCR analysis for cell expression of genes coding for integrin β1 (*ITGB1*) and E-cadherin (*CDH1*) on various membranes and well bottom surfaces (WBS). *GAPDH* was used as housekeeping gene, and its expression served as control.

To investigate the effect of PEG-modified membranes on cell adhesion proteins, HepG2 and HK-2 cells cultured for 144 h on various membranes were analyzed for expression of the genes coding integrin β1 (*ITGB1*) and E-cadherin (*CDH1*). HepG2 cells cultured on the three PEG-modified membranes showed weak expression of *ITGB1* and strong expression of *CDH1*, but cells cultured on PSf membranes/WBS showed the opposite expression patterns for the two genes (Fig. 7). HK-2 cells showed much lower *ITGB1* expression and higher *CDH1* expression on P123/F127 membranes than on PSf/L121/WBS. In regard to cell type, HK-2 renal tubular cells exhibited higher *ITGB1* and lower *CHD1* expression than HepG2 liver cells on each membrane.

**Figure 8:**
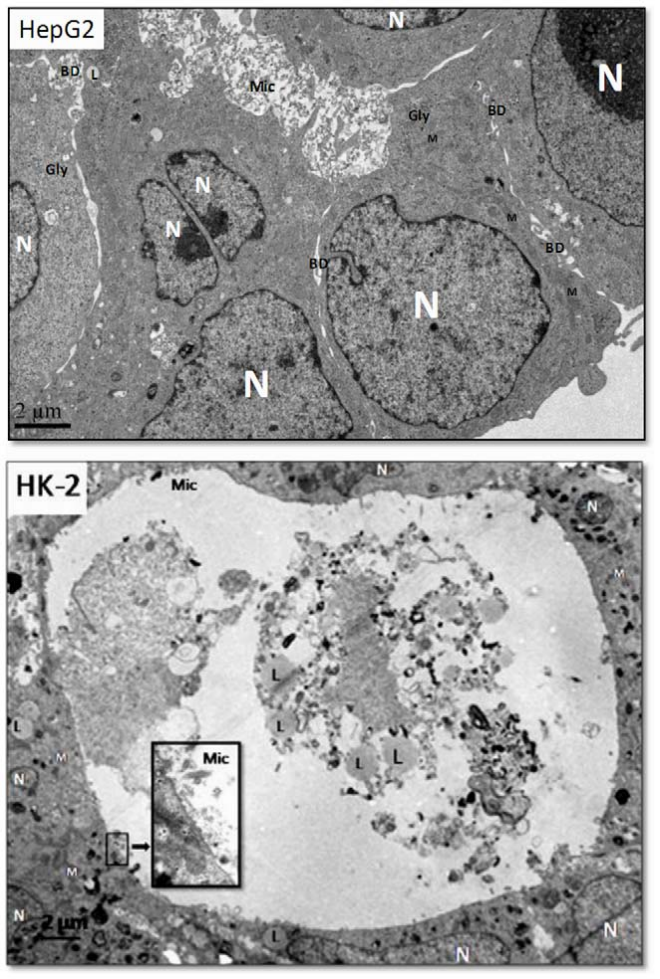
Ultrastructure of HepG2 and HK-2 cells cultured for 144 h on F127 membrane. nuclear (N), mitochondria (M), lipid droplet (L), glycogen particle (Gly), bile duct (BD), microvillus (Mic).

Since both liver and renal tubular cells self-assembled well on F127 membranes, HepG2 and HK-2 cell aggregates were selected for TEM observation (Fig. 8). As shown in Fig. 8A, HepG2 aggregates exhibited extensive cell-cell connections with abundant bile ducts, nuclei, numerous mitochondria, glycogen particles, and microvilli. The HK-2 renal tubular cells, in contrast, formed cysts surrounded by a cell layer. The cysts were about 30 µm in diameter, with microvilli on the inside surface and filled with cell fragments and lipid droplets (Fig. 8B).

## Discussion

This study explored the cytocompatible PEG-modified PSf membranes which could regulate the morphology and functions of epithelial cells in ways mimicking cell performance in vivo. Our findings are unique because they show the importance of the endogenous cytocompatibility, which has been neglected in the design of biomaterials.

Our results show that the self-assembly of liver cells was systematically influenced by modifying membrane surfaces with varying PEG content. With increasing PEG content of membrane surfaces, the morphology of HepG2 liver cells progressed from a confluent monolayer to elongated/flat aggregates and finally to round spheroids (Fig. 4). Differently, primary hepatocytes completely aggregated on surfaces, even with a low PEG content, e.g., the L121 membrane in this study and PEG_350_-grafted PSf membranes in our previous study [22], indicating that the morphology of primary cells was more readily influenced than that of a liver tumor cell line. Thus, our results demonstrate not only the influence of PEG membrane content on liver cell morphology, but also the differential performance of primary liver and tumor cells. In contrast, previous reports of liver cells forming spheroids on three-dimensional scaffolds [24], [25] and two-dimensional surfaces [7] only demonstrated the effect of a specific methodology on spheroid formation without exploring trends in cell behavior. Our study's systematic investigation of the effects of membrane PEG content on cell morphology, rather than describing a specific methodology, might facilitate the mechanistic investigation of cytocompatibility with biomaterials.

The membrane modification with PEG was, for the first time, found to facilitate self-assembly of renal tubular cells on flat surface. To date, renal cells in vitro formed tubular like structure [26] and have only been shown to form renal tubular cysts when entrapped within a hydrogel [27], [28]. The cyst structure found in our renal cells grown on high PEG-content membranes mimics the in vivo structure of renal tubules, which consists of an interior lumen lined with cuboidal renal tubular cells [29]. MDCK cells, another renal tubular cell line, showed a similar trend in cell self-assembly as HK-2 cells. We would like to mention that the renal tubular cells were less sensitive than liver cells to the PEG content of membranes. This lower sensitivity of renal tubular cells was due to their naturally stronger adhesive capability, which was confirmed by their higher expression of *ITGB1* (reflecting an integrin-mediated cell-substrate interaction [30]). As a whole, the simple surface modification by PEG may facilitate the formation of renal cysts and thus could benefit the development of potent renal assistive devices in the future.

An interesting common feature of PEG-modified PSf membranes, regardless of cell type, was their ability on sustaining the largely low proliferation and high level of cell-specific functions which were not found on PSf membrane/WBS. All three proliferating cell lines (HepG2, HK-2, and MDCK) showed much slower doubling times (≥6 days, estimated from Fig. 3A) in aggregates on F127 membranes than monolayer cultures on PSf membrane/WBS (about 2 days, estimated from Fig. 3A). Such largely reduced cell growth in aggregates was closer to the very low proliferation rate of liver cancer cells in vivo [17]. In contrast, the cell aggregates showed much higher levels of cell function than the monolayer cultures. For example, HepG2 aggregates on F127 membranes showed 2- to 5-fold higher liver-specific functions than HepG2 monolayer cultures on PSf membrane/WBS. Similar to our findings with HepG2 cells, liver cells are well known to express higher specific functions in aggregates than in monolayer [31], [32]. Nevertheless, such an impact of cell morphology on function has not been illustrated for renal tubular cells in vitro. Our study results provide the first evidence that aggregates of either HK-2 or MDCK renal cells display renal functions upregulated to much higher levels than in monolayer. As cell functions are normally downregulated after in vitro culture, the highly expressed cell functions in aggregates on PEG-modified membrane could, to some extent, reflect these cells' in vivo performance. Taken together, our results on both cell proliferation and functional expression suggest that cells might approach their in vivo performance more on PEG-modified membranes than on PSf membrane/WBS.

The surface modification by PEG might alter morphology/differentiation and gene expression of both types of liver and renal cells on flat membranes due to impaired cell-substrate (cell-membrane) interactions. Substrates are claimed to interact with cells via either non-specific adhesion or specific adhesion mediated by surface-adsorbed or -grafted ECM proteins and elements like RGD [5]. Another possibility is that substrate surfaces could be modified by adsorption of ECM proteins (such as fibronectin) in serum-containing medium. Our results on cell adhesion in FBS and protein-free medium show similar cell adhesion on PSf membranes/WBS regardless of medium, indicating the dominance of non-specific cell-surface interactions, while the largely different cell adhesion on PEG-modified membranes in the two mediums suggests the involvement of ECM-mediated specific adhesion as FBS was required for such cell adhesion. Furthermore, PEG, a well known bioinert molecule lacking any interaction with cells [33], covered the PSf membrane surface and more likely impaired non-specific cell adhesions on PSf membranes, making specific adhesion via ECM adsorption more dominant. Lastly, the downregulated gene expression of *ITGB1* (reflecting cell-substrate interactions [30]) and upregulated gene expression of cadherin *CDH1* (reflecting cell-cell interactions [30]) of cells on PEG-modified membranes suggest that the non-specific cell adhesion occurred between cells and the hard surface of membranes while the ECM-mediated specific cell adhesion occurred between two cells. Thus, PEG modification of membranes seemed to decrease cell-substrate interactions and increase cell-cell interactions.

The PEG modification on membranes resulted in an inhibited non-specific cell adhesion, which should be a good approximation of the in vivo specific interactions between cells and the ECM [34]. Surface modifications with ECM (e.g., fibronectin [33]) or its elements (e.g., RGD [35]), though having been extensively used in cell culture, only enhanced cell adhesion/spreading instead of promoting or facilitating cell assembly. It seems that the modification of surfaces by ECM or its elements made cells performing quite different from that on PEG-modified surfaces. It could be possible that the excessively modified ECM or its elements on surfaces might result in a largely enhanced cell-substrate binding and reduced cell-cell interactions. Based on the morphology and functional expression of epithelial cells on PEG-modified membranes, we would like to modify the concept of cytocompatibility by emphasizing that biomaterials should promote the ability of cells to sustain or adopt in vivo morphology and functions as much as possible. In this respect, many biomaterials might not achieve cytocompatibility as measured by extensive cell spreading/adhering/rapid proliferation because of a critical deviation from the in vivo situation. It is also likely that tissue culture plates, which are widely used in cell culture because most cells adhere to and spread on their surface, will not provide good cytocompatibility.

In conclusion, PEG modification on PSf membranes promoted liver and renal tubular cells to aggregate into organ-like structures. Such surface modification possibly impaired cell-membrane interactions and increased cell-cell interactions. All four liver/renal tubular cell types on F127 membranes with the proper PEG content displayed the highest cell functions and were closely related to their liver/renal tubule-like ultrastructure in vivo. In these aspects, the cells cultured on PEG-modified PSf membranes closely approximated cells in vivo, suggesting that these membranes are highly cytocompatible.

## Materials and Methods

### Materials

PSf (average MW 65000) was provided by Shuguang Chemical Factory (Shanghai, China). Pluronics L121, P123 and F127 (detailed information in Table 2), 5-carboxyfluorescein diacetate (5-CFDA, AM), DMEM medium (low glucose), collagenase (type IV), insulin, dexamethasone and glucagon were purchased from Sigma-Aldrich Chemical Company (St. Louis, MO, USA). L-glutamine, penicillin and streptomycin were purchased from Amresco Inc. (Solon, Ohio, USA). Fetal bovine serum (FBS) was obtained from Hangzhou Sijiqing Biological Eng. Material Co., Ltd. (Hangzhou, China). RNA isolation kit, first strand cDNA synthesis kit, and PCR reagents were purchased from Takara Bio. Inc. (Otsu, Japan). Primers were synthesized by SBS Genetech Co., Ltd (Shanghai, China). The rat and human albumin ELISA quantitation kits were purchased from BETHYL Laboratories, Inc. (Montgomery, TX). The L-γ-glutamine-4-nitroanilide colorimetric kit was purchased from Saike Bio. Inc. (Ningbo, China). The remaining chemicals were obtained from local chemical suppliers and were all of reagent grade.

**Table 2:** Chemical structure of Pluronics.

*Pluronic*	*Total MW*	*Content of PEG (wt%)*	*MW of PEG*	*MW of PPO*	*Average number ^a^*		*HLB value*
					n	m	
L121	4400	10	210×2	3980	5	68	1–7
P123	5800	30	880×2	4040	20	70	7–12
F127	12600	70	4410×2	3780	98	67	18–23

Note. a: 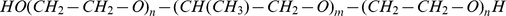. PPO: polypropylene oxide; PEG: polyethylene glycol; HLB: hydrophilic-lipophilic balance.

### Membrane preparation and characterization

Ultrafiltration PSf membranes were prepared with or without Pluronic blending by the immersion precipitation method at room temperature [22]. According to the casting composition in Table 1, PSf and Pluronics (L121, P123, and F127) were dissolved in N,N-dimethylacetamide (DMAc) to make 18 wt% polymer solution, while the pure PSf solution was prepared with only 18 wt% of PSf dissolved in DMAc. After overnight incubation without stirring at room temperature, the solutions were cast on clean glass plates to a predetermined thickness with a Gardener knife at 25°C, and immediately immersed in a bath of deionized water at 25°C. The formed membranes were kept in deionized water for at least 24 h until use.

The hydrophilicity of the membrane surface was characterized by its static water contact angle using the sessile drop method with a contact angle goniometer (OCA20, Dataphysics Instruments with GmbH, Germany) at 25°C and 40% relative humidity.

X-ray photoelectron spectroscopy (XPS) analysis was used to determine the fraction of PEG in the near surface region of the membranes. Rectangular pieces were cut from each membrane and dried. Experiments were performed on a VG ESCALAB MARK II X-ray photoelectron spectrometer (VG Scientific LTD, UK) employing a monochromatic Mg Ka source (1253.6 eV) and an electron takeoff angle of 90° relative to the sample plane. A survey scan of 0–1100 eV binding energy range and a high-resolution scan of C 1 s peak were run for each sample. The percentage of –C-C- and –C–O– bonds was calculated using the peak area at 283.4 eV and 284.8 eV in peak of carboxyl groups.

### Cell culture

All animal procedures were carried out in accordance with the Guide for the Care and Use of Laboratory Animals by the United States National Institutes of Health. The study was approved by the Ethical and Research Committee of Zhejiang University, China. Rat hepatocytes were isolated from the livers of male Sprague-Dawley rats (200–250 g) by the two-step collagenase perfusion method [36]. Hepatocytes with viability greater than 85%, as assessed by Trypan blue exclusion, were used.

HepG2, MDCK, and HK-2 cells were from a commercial source of the American type culture collection (ATCC) and cultured in DMEM medium (low glucose) supplemented with 10% FBS, L-glutamine 2 mM, and glucose 1 g/L. Confluent cells were trypsinized, counted by hemacytometer, and centrifuged. The resulting cell pellets were re-suspended in fresh medium and seeded at 1×10^5^ cells/ml.

**Figure 9:**
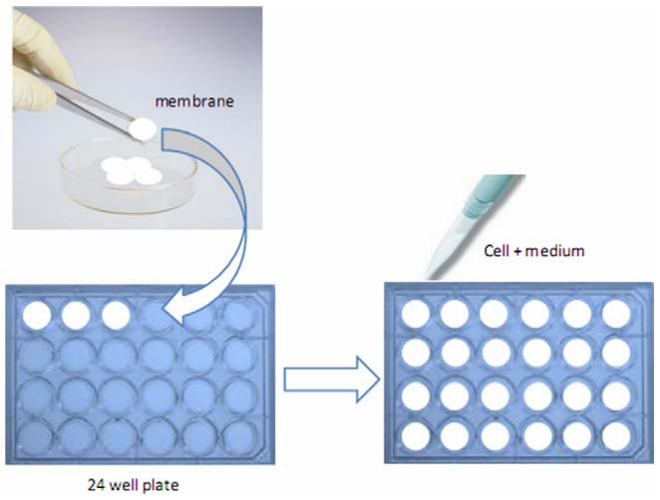
Preparation scheme for cell culture on polyethylene glycol-modified polysulfone membranes.

The procedure for culturing cells on membranes is illustrated in Fig. 9. Briefly, the membranes were cut into small round pieces to match the size of wells in a 24-well plate and placed on the well bottom surface. Well bottom surfaces (WBS) without membranes served as controls. Primary cultures of freshly isolated rat hepatocytes were seeded at a density of 2×10^5^ cells/well with DMEM medium (low glucose) supplemented with L-glutamine 2 mM, penicillin 100 U/ml, streptomycin 100 µg/ml, dexamethasone 100 nM, insulin 0.2 U/ml, glucagon 4 ng/ml and 5% FBS. HepG2, HK-2 and MDCK cells were seeded at density of 1×10^5^ cells/well with DMEM medium (low glucose) supplemented with L-glutamine 2 mM, glucose 1 g/L, and 10% FBS. All cells were cultured at 37°C in a humidified atmosphere containing 5% CO_2_.

### Cell adhesion and proliferation

Rat hepatocytes and HepG2/HK-2/MDCK cells were suspended in medium with 10% FBS or protein-free medium, plated onto membranes or well bottom surfaces at 1×10^5^ and 5×10^4^ cells/well, and incubated for 4 h at 37°C. Then the membranes and wells were carefully rinsed with phosphate buffered saline (PBS) to remove unattached cells. The membranes/WBS were then immersed in trypsin-EDTA (0.25% vs 0.02%) solution to lift the adhered cells. After incubation for 20 min, the trypsin-EDTA solution was neutralized with culture medium. The cell numbers on membranes and WBS were counted for triplicate samples using a hemacytometer. The percent of attached cells was calculated as follows:
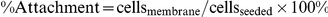



After 48, 96, or 144 h of culture, cells were isolated from membranes or WBS by trypsinization with trypsin-EDTA (0.25% vs 0.02%) solution, washed, and viable cell number were determined with a hemocytometer using trypan blue dye exclusion test [37]. Direct cell counts were performed in triplicate.

### Observation of cell morphology

The morphology of cells after 144 h culture on membranes was observed by scanning electron microscope (SEM). Briefly, the cells and membranes were fixed overnight in 2.5% glutaraldehyde solution. The sample was washed three times with PBS and dehydrated by a graded series of ethanol (50%, 70%, 80%, 90%, 95% and 100%) for 15 to 20 minutes at each step, transferred to pure isoamyl acetate for 1 h, and dehydrated in Hitachi Model HCP-2 critical point dryer with liquid CO_2_. The dehydrated specimen was coated with gold-palladium and observed in SEM (TM-1000, Hitachi, Japan).

The ultrastructure of HepG2 and HK-2 cells after 144 h culture on F127 membranes was detected by transmission electron microscope (TEM). The cells and membranes were fixed overnight in 2.5% glutaraldehyde solution and then post-fixed for 1 h in 2% OsO_4_. Samples were subsequently dehydrated by graded ethanol (50%, 70%, 80%, 90%, 95% and 100%) and embedded in epoxy resin. Thin sections were cut orthogonally to the cell layer and stained with uranyl acetate before imaging by TEM (JEM-1200EX, JEOL, Japan).

### Gene expression of adhesion proteins

Total RNA was isolated from HepG2 and HK-2 cells after 144 h incubation on both membranes and WBS using trizol reagent. The quality of RNA was assessed by sample absorbance at 230, 260, and 280 nm. cDNA was synthesized as previously described [38] and amplified by the polymerase chain reaction (PCR), while tubes containing no template were used as negative controls. Gene expression was measured for the adhesion proteins, integrin β1 (*ITGB1*) and E-cadherin (*CDH1*), while expression of glyceraldehyde-3-phosphate dehydrogenase (*GAPDH*) was used as a control. The primers are listed in Table 3.

**Table 3:** Primers for *GAPDH*, *ITGB1,* and *CDH1*.

*Gene*	*NCBI Reference Sequence*	*Forward primer (5′-3′)*	*Reverse primer (5′-3′)*	*Product size (bp)*
*GAPDH*	NM_002046	TCACCAGGGCTGCTTTTAAC	TGTGGTCATGAGTCCTTCCA	479
*ITGB1*	NM_002211	ACTCAGATCCAACCACAGCA	ACATTCCTCCAGCCAATCAG	476
*CDH1*	NM_004360	GGATGTGCTGGATGTGAATG	TTGGGTTGGGTCGTTGTACT	517

### Assay of hepatic functions

Hepatic functions of HepG2 cultured cells and primary rat hepatocytes were assessed by urea synthesis, albumin secretion, and cytochrome P450 activity. Urea and albumin content in culture medium was determined by urea nitrogen kit and albumin ELISA assay kit [22], respectively. Cytochrome P450 (CYP) 1A2 and 3A activities were assayed to represent CYP 450 activities. After 144 h of culture, HepG2 and rat hepatocytes were immersed in 500 µl PBS containing 50 µM phenacetin or 250 µM testosterone, respectively. After 3 h of incubation at 37°C, 250 µl of acetonitrile was added to the solution, which was transferred to new tubes. The metabolites of phenacetin and testosterone in solution, represented by acetaminophen and 6β-hydroxy testosterone, respectively, were analyzed by Varian ProStar 210 Series HPLC (Walnut Creek, CA, USA) equipped with a ChromSpher C18 column (150 mm×4.6 mm, 5 µm particle size). The mobile phases, at flow-rate of 1 ml/min, were methanol: water (50∶50) for acetaminophen detection and methanol: NaH_2_PO_4_ (20 mM): triethyl-amine (50∶50∶0.1), pH 6.3 for 6β-hydroxy testosterone detection with an absorbance peak at 254 nm. The CYP 1A2 and 3A activities of HepG2 and rat hepatocytes were represented by the formation rate of acetaminophen and 6β-hydroxy testosterone, respectively.

### Assay of renal functions

Renal functions of HK-2 and MDCK cells were assessed by alkaline phosphatase, and γ-glutamyltransferase, and multidrug resistance-associated protein 2 (MRP2) activities. Alkaline phosphatase activity was measured as reported [39]. Briefly, HK-2 and MDCK cells were lysed in 1% Triton X-100 after twice washing with PBS, and 20 µl of lysis solution was added to 100 µl reaction solution containing 0.5 M Tris, 10 mM p-nitrophenyl phosphate disodium, 2 mM MgCl_2_, and 1 mM ZnCl_2_ (pH 9.8–10). After incubation at 37°C for 30 min, the reaction was terminated by adding 80 µl NaOH (1 M), and the absorbance of the solution at 450 nm was read by a microplate reader. Similarly, γ-glutamyltransferase activity was assayed using a L-γ-glutamine-4-nitroanilide colorimetric kit [40].

The activity of MRP2, which is involved in excreting small organic anions from proximal renal tubule endothelial cells, was represented by the efflux of 5-carboxyfluorescein diacetate (5-CFDA) in the presence or absence of the MRP2 inhibitor, probenecid [41]. Briefly, HK-2 and MDCK cells were initially loaded with 10 µM 5-CFDA for 30 min and washed twice in ice-cold (0°C) Hank's balanced salt solution (HBSS) to terminate dye loading. Subsequently, cells were incubated in 500 µl of dye-free HBSS for 30 min at 37°C in the presence or absence of 1 mM probenecid. The efflux of 5-CFDA from cells into HBSS was measured by fluorescence spectroscopy at 485/585 nm. Mrp2 activity was represented by the differential efflux of 5-CFDA in the presence or absence of probenecid.

### Data analysis

All data from cell experiments were analyzed by means ± SD from three independent experiments. Comparisons between multiple groups were performed with the ANOVA test by SPSS. *P*-values less than 0.05 were considered statistically significant.
